# Nonlinear similarity characterisation and validation of dynamic response of ship stiffened plate structure under explosion load impacts

**DOI:** 10.1038/s41598-024-66366-6

**Published:** 2024-07-09

**Authors:** Dongyan Shi, Jiuqiang Wang, Zhikai Wang, M. K. Helal Wasim

**Affiliations:** 1 College of Mechanical and Electrical Engineering, Harbin Engineering University, Harbin, 150001 China; 2 College of Ship Engineering, Harbin Engineering University, Harbin, 150001 China; 3 Mechanical Engineering Department, Kafr El-Sheikh University, Kafr El-Sheikh, 33156 Egypt

**Keywords:** Underwater explosion, Plate frame structure, Impact nonlinearity, Hurst index, Similarity law, Engineering, Applied physics

## Abstract

The similarity test of ship stiffened plate structures under underwater explosions is a cost-effective and efficient method to evaluate the vitality of ships and guide the design of their shock resistance. This study focuses on the nonlinear impact response model tests of ship stiffened plate structures and their similarity laws with actual ships. The vertical motion of the ship stiffened plate structure is characterized by the Hurst index, and an equivalent relationship between the Hurst index of the model and the prototype is derived from classical similarity law. Based on the Hurst index, a similarity transformation relationship between the strain signals of the model and prototype is established. To verify the conclusions, similarity experiments of underwater explosions were conducted on both the model and the prototype. The original signals were grouped by the natural vibration period to determine the variation of the Hurst index over time. The model experiment strain signals for each natural vibration period were converted and compared with the prototype experiment results to verify the method's effectiveness. Simultaneously, the Hurst index of the stiffened plate structure under explosive shock load and its similarity transformation relationship with the prototype were simulated and analyzed. This provides theoretical and technical support for conducting analogous nonlinear response experiments for ship underwater explosions.

## Introduction

As the competition for maritime equipment among countries becomes increasingly prominent, countries around the world are competing to develop advanced underwater weapons with high speed, large dose, and precise guidance, which poses a huge threat to the vitality and combat effectiveness of ships. Theoretical and experimental research on the damage mechanisms and impact responses of ship structures and scale models under different underwater explosion loads plays a key role in improving the explosion resistance and shock performance of ships.

Conducting full-scale experiments is expensive in terms of both economy and time. Therefore, in experimental design, small-scale model experiments are used to predict the impact response of full-scale structures, thereby reducing economic costs, shortening the experimental cycle, and simplifying the experimental environment. The underwater explosion shock wave has a high peak value and short pulse width, which can cause serious damage to the ship structure. Similarity methods are widely used in the field of impact dynamics^1–3^. How to accurately predict the dynamic response of a structure under the action of underwater explosion using similarity methods has been a hot topic of concern for scholars around the world^4–7^. Similarity experiments are a method that scales down the experimental object. In scale-down similarity experiments, researchers scale down the size, shape, and characteristics of the experimental object by a certain proportion to obtain a smaller model with similar properties and behaviors. In the study of ship stiffened plate structures, when conducting dynamic response scale-down experiments under impact load, the experimental object is called a prototype or large model, having the same size as the actual ship stiffened plate structure. The size of the experimental object is proportionally reduced to the actual size of the ship's stiffened plate structure, which is called a model or small model.

Scholars have conducted a large number of studies to solve the similarity problem in the field of underwater explosion shock response. Su Qiu^8^ and other scholars used dimensional analysis to derive an empirical formula similar to that of Cole's for underwater explosions, and ultimately verified the accuracy of this similarity law through experiments. Lu^9–12^ and other scholars also conducted numerical simulations (including similarity theory analysis, π theorem derivation, etc.) on different targets, and verified the conclusion that the shock dynamic response characteristics of the prototype and model structures during underwater explosions are basically the same. The above scholars only considered linear characteristics, and few scholars considered nonlinear characteristics. Wang Peng^13^ believes that classical similarity theory has theoretical flaws in the nonlinear field, and considers nonlinear similarity when deriving the similarity relationship between prototypes and models. Pei and other scholars^14^ verified that the response of the model in the nonlinear and linear stages can be equivalent towards the prototype direction by considering nonlinear factors such as the slenderness ratio of the slats and the flexibility coefficient of the stiffeners. Wang^15^ proposed a similarity transformation method between the model and prototype based on the shear nonlinear characteristic parameters of the ship stiffened plate, and verified the effectiveness of this shear nonlinear similarity method. Igor^16^ and other scholars considered nonlinear factors such as the ultimate strength of materials, scaled the catamaran cabin section equivalently, and verified through numerical and experimental methods that the sagging limit bending moment of the model can be converted to the actual cabin.

Scarlat et al. studied nonlinear similarity patterns in daily exchange rate time series based on the Hurst index. Exchange rate changes follow an infinitely scalable curve, and there is no model experiment to provide a transformation for similarity research. Therefore, only nonlinear self-similarity characteristics can be studied^17^. Similarly, Hu et al.^18^ introduced the concept of fractal and used the Hurst index as a characterization parameter to study the dynamics and behavior of nonlinear financial time series in the Russian market. Liu, Y et al., Liu, XL et al., Huang, XM et al. studied nonlinear similarity patterns in various fields such as energy use, artificial DNA sequences, and mathematical Fibonacci sequences based on the Hurst index. However, there is still a gap in the similarity law of nonlinear shock response^19–22^.

When subjected to underwater explosion loads, ship structures undergo complex nonlinear dynamic responses, characterized by rapid and significant transmission of large impact loads in a short period of time. This phenomenon falls under the category of strongly nonlinear problems. Currently, when considering underwater explosion model experiments, the design of similarity criteria is mainly based on dimensional analysis. However, in this process, the influence of nonlinear factors is often neglected. This article innovatively breaks through the limitations of traditional similarity theory and uses the nonlinear characteristic parameter—Hurst index to describe the similarity law of nonlinear impact response between model and prototype ship stiffened plate structures. Based on Hurst index, a multi-parameter similarity transformation rule including strain signals is derived. The derived rule is verified based on experimental and simulation results. Compared with traditional similarity rules, the proposed method in this article has lower error.

### Model

According to literature 21, the necessary and sufficient condition for the similarity of two phenomena is that the values of the dimensionless combination set that constitutes the foundation are equal to constants. The basis of mechanical similarity in an elastic equilibrium state is the following three dimensionless variables: $\sigma , \frac{E}{\rho gB}, \frac{P}{E{B}^{2}}$.

Based on the above three dimensionless variables, *g* = const and *B* = const. When *g* is a constant, experiments cannot be conducted. Currently, prototype and model experiments on the impact response of ship stiffened plates under underwater explosion loads cannot be conducted in centrifuges. Based on dimensional analysis, a dimensional matrix of relevant physical quantities in a similar system is established based on the second law of similarity, and the similarity coefficients of relevant physical quantities are derived and obtained:

1 $$f(\rho ,\mu ,E,v,a,P,A,\sigma ,\varepsilon ,\dot{\varepsilon },F,L,T)=0$$

where $\rho$ is the material density;$\mu$ is Poisson ratio; *E* is the modulus of elasticity; and *v* is the peak velocity along the z-axis during deformation; *P* is the peak intensity of the shock wave in the water; *A* is the loaded cross-sectional area; $\sigma$ is the yield stress; $\varepsilon$ is the strain; $\dot{\varepsilon }$ is the strain rate; *F* is the z-direction impact load under the action of the underwater explosion shock wave; *L* is the size of the structure; *T* is the impact time. The geometric similarity coefficient (shrinkage ratio) of the model during the experiment is $\lambda$. Taking *L*, *T* and *F* as the basic quantities, the expressions for the quantities of the relevant physical quantities are obtained as shown in Table 1.

**Table 1:** Expressions of the magnitudes of the relevant physical quantities of the similar model.

Physical quantity	Symbol	Dimension
Material density	*ρ*	$F{L}^{-4}{T}^{2}$
Poisson ratio	$\mu$	${F}^{0}{L}^{0}{T}^{0}$
Elasticity modulus	$E$	$F{L}^{-2}{T}^{0}$
Speed	$v$	${F}^{0}L{T}^{-1}$
Accelerated Speed	$a$	${F}^{0}L{T}^{-2}$
Shock wave intensity	$P$	$F{L}^{-2}{T}^{0}$
Axial cross-sectional area	$A$	${F}^{0}{L}^{2}{T}^{0}$
Yield stress	$\sigma$	$F{L}^{-2}{T}^{0}$
Strain	$\varepsilon$	${F}^{0}{L}^{0}{T}^{0}$
Strain rate	$\dot{\varepsilon }$	${F}^{0}{L}^{0}{T}^{-1}$
Axial impact loading	$F$	$F$
Geometric length of terms	$L$	$L$
Attack time	$T$	$T$

Ten π items can be obtained:

2 $${\pi }_{1}=\frac{\rho {L}^{4}}{F{T}^{2}},{\pi }_{2}=\mu ,{\pi }_{3}=\frac{E{L}^{2}}{F},{\pi }_{4}=v\frac{T}{L},{\pi }_{5}=a\frac{L}{T},{\pi }_{6}=P\frac{{L}^{2}}{F},{\pi }_{7}=A\frac{1}{{L}^{2}},{\pi }_{8}=\sigma \frac{{L}^{2}}{F},{\pi }_{9}=\varepsilon ,{\pi }_{10}=\dot{\varepsilon }T$$

Therefore:

3 $${\pi }_{9}=\varepsilon =f({\pi }_{1},{\pi }_{2},{\pi }_{3},{\pi }_{4},{\pi }_{5},{\pi }_{6},{\pi }_{7},{\pi }_{8},{\pi }_{10})$$

According to the Rescaled Range Analysis method (R/S analysis for short), R is the range of each subsequence, and S is the standard deviation of each subsequence. For both the prototype and the model, there is an initial moment $t=T=0$, then there is the following relationship between the extreme deviation and the standard deviation of the prototype and the model:

4 $$\frac{R}{S}=\frac{\frac{1}{\lambda }\widehat{R}}{\frac{1}{\lambda }\widehat{S}}=\frac{\widehat{R}}{\widehat{S}}={\left(\frac{\tau }{2}\right)}^{\widehat{H}}$$

where* R* represents the range of prototype response; *S* represents the mean square deviation of the prototype response; $\widehat{R}$ represents the extreme deviation of model response; $\widehat{S}$ is the mean square deviation of the model response; and τ is the total action time of the prototype response. That is to say, the Hurst index of prototype and model are equal:

5 $$H=\widehat{H}$$

If only the vertical impact response of the ship's stiffened plate structure is considered, the ship's impact environment can be simplified to one-dimensional nonlinear unsteady Brownian motion, where the vertical response of the prototype's stiffened plate structure is set to *X*(t) and the vertical response of the model's stiffened plate structure is set to *x*(t), both of which follow the random walk model. If the geometric similarity between the prototype and the model is fully satisfied, then under the conditions of conventional similarity in explosion distance and charge size, the working conditions satisfy the similarity of excitation loads, and the boundary conditions are similar (i.e., the boundary motion conditions are similar, and displacement and velocity satisfy the similarity law). Then there should be:

6 $$x\left(t\right)=\lambda X\left(T\right)$$

Let ${x}_{i}=x\left({t}_{i}\right),{X}_{i}=X\left({T}_{i}\right)\left(i=\text{0,1},2,...,N\right)$, which is given by Eq. (6):

7 $${x}_{i}=\lambda {X}_{i}$$

Equation (7) is a motion similarity law introduced by the law of geometric similarity, namely the scale ratio. However, in practical engineering, due to the nonlinear characteristics of the dynamic response of ship stiffened plate, the excitation loads and boundary conditions of the prototype and model at a certain location exhibit uncertainty. Therefore, for complex stiffened plate structures of ships, it is difficult to ensure that the motion similarity ratio of Eqs. (6) and Eqs. (7) is consistent.

The position change of Brownian particles follows the following power law:

8 $$\langle {\left[X\left(T\right)-X\left({T}_{0}\right)\right]}^{2}\rangle =2D\tau \frac{T-{T}_{0}}{\tau }\propto {\left(\frac{T-{T}_{0}}{\tau }\right)}^{2H}$$

where *D* is the diffusion coefficient, and Mondro refers to the power-law non-regular motion that obeys the above equation as fractional Brownian motion (abbreviated as FBM), where the exponent *H* is not equal to 1/2, but takes values in (0, 1). The position *X*(*t*) of the formula particle is represented by *B*_*H*_(*t*), and this random function is called the Brownian function. Therefore:

9 $${B}_{H}\left(T\right)-{B}_{H}\left({T}_{0}\right)=X\left(T\right)-X\left({T}_{0}\right)\propto {\left|T-T\right|}^{H}$$

Fractional Brownian motion has the following scalar relationship^21^:

10 $${B}_{H}\left(\lambda T\right)-{B}_{H}\left(0\right)={\lambda }^{H}\left[{B}_{H}\left(T\right)-{B}_{H}\left(0\right)\right]$$

Based on the invariance of the scalar (self-similarity) of the fractional Brownian motion, it can be concluded that:

11 $$\left\{\begin{array}{c}{B}_{H}\left(T\right)\propto {T}^{H}\\ {B}_{H}\left(\lambda T\right)={\lambda }^{H}{B}_{H}\left(T\right)\end{array}\right.$$

The mean square response of the model motion, $\left\langle {x^{2} } \right\rangle$, is:

12 $$\langle {x}^{2}\rangle ={\int }_{-\infty }^{\infty }{x}^{2}p\left(x\right)dx$$

In the formula, $p\left(x\right)=p\left(x,{\tau }_{x}\right)$, $x$ is the step length of the model random walk, and ${\tau }_{x}$ is the time taken to walk *N* steps. ${\tau }_{x}$ is assumed to be deterministic. When ${\tau }_{x}$ takes a specific value, the corresponding model mean-square response ($\left\langle {x^{2} } \right\rangle$) is:

13 $$\begin{aligned} & \left\langle {x^{2} } \right\rangle = \int\limits_{ - \infty }^{\infty } {x^{2} } p\left( x \right)dy = \int\limits_{ - \infty }^{\infty } {\left( {\lambda^{H} X} \right)}^{2} p\left( {\lambda^{H} X} \right)\lambda^{H} dX \\ & \quad = \int\limits_{ - \infty }^{\infty } {\lambda^{2H} } X^{2} \lambda^{ - H} p\left( X \right)dX \\ & \quad = \lambda^{H} \int\limits_{ - \infty }^{\infty } {X^{2} p\left( x \right)dX} \\ & \quad = \lambda^{H} \left\langle {X^{2} } \right\rangle \\ \end{aligned}$$

Comparing Eq. (7) with Eq. (13), it can be noted that in the classical similarity law, $y=\lambda x$ In the similarity law of fractional Brownian motion, there is a significant difference between the two, $x\sim \sqrt{\langle {x}^{2}\rangle },X\sim \sqrt{\langle {X}^{2}\rangle }$ , that is $x={\lambda }^{H}X$. For the classical similarity law:

14 $$X=\frac{1}{\lambda }x=\frac{L}{l}x$$

The similarity law of fractional Brownian motion^23^:

15 $$\sqrt{\langle {X}^{2}\rangle }=\frac{1}{{\lambda }^{H}}\sqrt{\langle {x}^{2}\rangle }={\left(\frac{l}{L}\right)}^{H}\sqrt{\langle {x}^{2}\rangle }$$

Based on the equation of normal strain:

16 $$\varepsilon =\underset{P\to 0}{lim}(\frac{\Delta P}{P})$$

where $P$ is the length before deformation, that is stiffened plate structure size,$\Delta P$ is the elongation, given by $\sqrt{\langle {X}^{2}\rangle }$ obtain:

17 $$\frac{\sqrt{\langle {X}^{2}\rangle }}{\sqrt{\langle {x}^{2}\rangle }}\cdot \frac{L}{l}=\frac{{\varepsilon }_{p}}{{\varepsilon }_{m}}={\lambda }^{H-1}$$

### Experimental design

**Figure 1:**
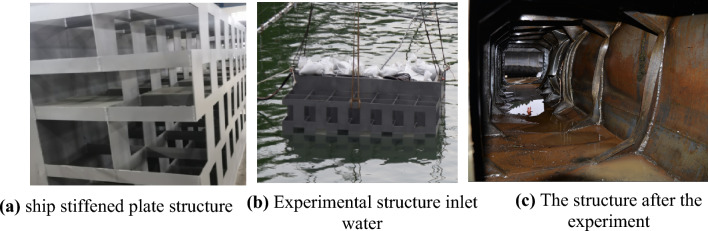
Physical drawing of the segment structure.

**Figure 2:**
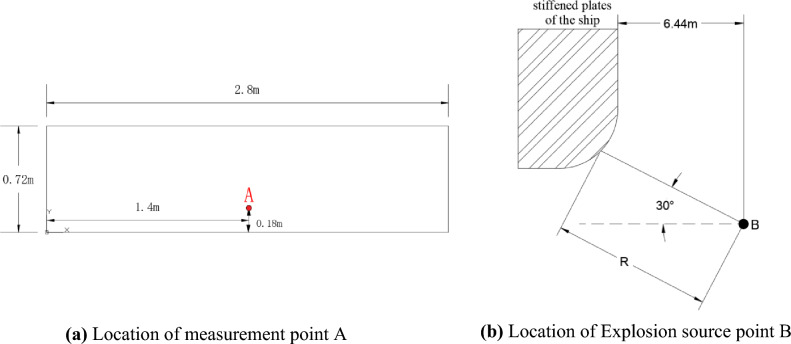
Measurement point location and explosion location.

To verify the correctness of the above conclusions, prototype and model underwater explosion experiments were conducted in a pool. The datasets used and/or analysed during the current study are available from the corresponding author on reasonable request. The structures of the prototype and model are completely identical, and the dimensions of each corresponding part fully meet the scale ratio requirements (both materials are Q345 steel). The ship stiffened plate structure used in the experiment is shown in Fig. 1a, with a scale ratio set to 1.5:1, and the external dimensions of both are shown in Table 2. The experimental process is shown in Fig. 1b, and the structure of the ship's stiffened plate after the experiment is shown in Fig. 1c. The experiment was conducted in a pool with a diameter of 40 m and a depth of 25 m. The surface of the structure was treated with three methods, namely removing oxidation and rust on the surface of the structure, sandpaper polishing and grinding, and alcohol wiping. After surface treatment, strain gauges were pasted on the surface of the structure with quick-drying glue. During the pasting process, bubbles in the pasting layer were squeezed out to ensure waterproofness of the strain gauges and data cables. The location of measurement point A is shown in Fig. 2a, and the relative position between the explosion source and the ship's reinforced plate structure is shown in Fig. 2b, where point B is the explosion source.

**Table 2:** Exterior dimensions of cabin structure.

Overall dimensions	Long (m)	Wide (m)	Height (m)
Prototype	4.2	1.7	2.4
Model	2.8	1.13	1.6

The measurement point A is arranged on the left bulkhead. The structural dimensions of each part of the model bulkhead are shown in Table 3. The prototype dimensions are designed according to Eq. (1), which means that the prototype's structural dimensions are 1.5 times those of the model. The working conditions of the prototype and model are shown in Table 4, which are set with reference to Eq. (1).

**Table 3:** Structural dimensions of frame.

	Thickness of plating	Floor thickness	Floor spacing	Width of floor	Dimensions of the rib profile
Upper deck	3mm	3mm	0.4m	83mm	33mmx3mm
Lower deck	3.75mm			\	\
Port bulkhead	3mm			92mm	37mmx3mm
Planking	3.75mm			100mm	42mmx3mm

**Table 4:** Working condition settings of prototype and model.

Measure point	Measurement target	Explosion distance (m)	Explosive mass (kg)
A	Prototype	12.93	15.5
	Model	8.62	4.6kg

### Hurst index validation and trend analysis

**Figure 3:**
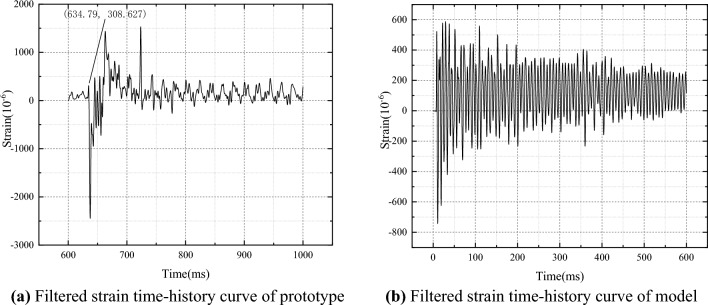
The filtered strain time-history curve.

The stiffened plates of the ship undergo elastic deformation, and the experiments are filtered using a low-pass filter with a cutoff frequency of 300 Hz ^24^. According to empirical equations, the natural frequency of the prototype is calculated to be 55 Hz, and the natural frequency of the model is calculated to be 80 Hz^25^. The filtered strain time history curve is shown in Fig. 3.

**Figure 4:**
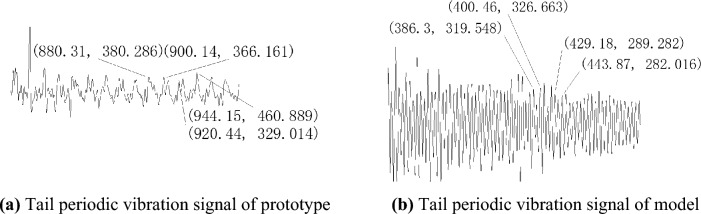
The filtered strain time-history curve.

The impact response of the ship's stiffened plate structure is divided into three stages: forced vibration under the action of shock waves, a transition stage from forced vibration to free vibration at the natural frequency, and free vibration at the natural frequency. As shown in Fig. 3a, the 600–634.79ms signal is irrelevant, and the first stage of the prototype is approximately 634.79-700ms, the second stage is approximately 700-800ms, and the third stage is approximately after 800ms. The third stage signal is an approximate periodic signal. Using a steady-state analysis method, this segment of the signal is captured to obtain Fig. 4a. From the figure, it can be seen that the stable vibration period is approximately 20ms and the natural frequency is approximately 50Hz. Similarly, from Fig. 4b, it can be seen that the stable vibration period of the model is approximately 14ms and the natural frequency is approximately 71Hz.

**Figure 5:**
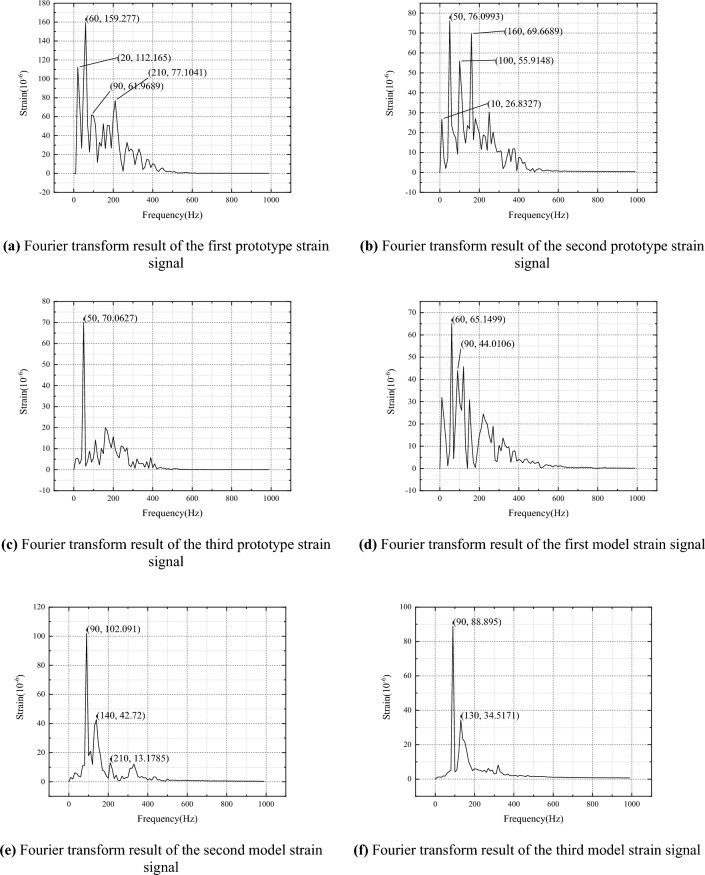
Fourier transform result.

The Fourier transform is performed on the three stages of the prototype and model, respectively, and the results are shown in Fig. 5.

Based on the Fourier transform results, combined with empirical formulas and free vibration analysis results, it can be inferred that the natural frequency of the prototype is approximately 60Hz, and the natural frequency of the model is approximately 90Hz. The natural periods of the prototype and model are 0.01666s and 0.01111s, respectively, with each natural period containing 1666 and 1111 sets of data. In order to analyze the nonlinear characteristics of the response signal through changes in Hurst index, the filtered raw data is grouped according to natural periods, with several data segments in each group. The Hurst indices of these data segments are calculated. This article uses two data grouping methods. Scheme 1: Each group of data consists of the previous group of data plus several new data sets until all data are included, that is, the amount of data in each group increases. Scheme 2: Each group has the same amount of data, equal to the amount of data within the natural period.

**Figure 6:**
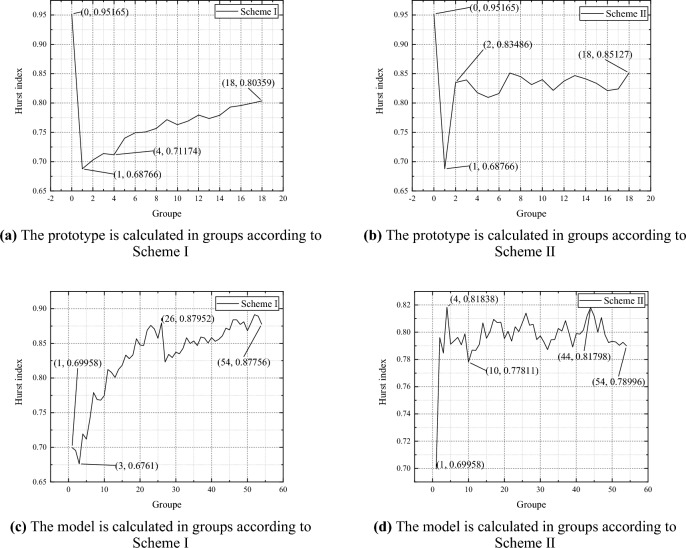
Calculation results of Hurst index.

The Hurst index calculation results are shown in Fig. 6, where the initial value of the Hurst index for the prototype strain is 0.95165, which is due to the fact that the strain data is an irrelevant signal within the range of 600–634.79ms. The rest of the Hurst index varies within the range of 0.68–0.88. According to literature 26, it is known that the larger the Hurst index, the stronger the regular directional (diffusive) motion of particles relative to irregular noise motion. According to Fig. 6a,c, the trend of regular directional (diffusive) motion of particles increases. Mondro said that fractional Brownian motion with a score of 0.5 < H < 1 is persistent motion, which means that the regular motion of particles is relatively strong, and the randomness (noise) is weak, and their trajectories are less tortuous, so they rarely self-intersect and are difficult to return to the original starting point. This means that the "density" of trajectories is very small, making it difficult to fill two-dimensional planes or three-dimensional spaces, but it is more conducive to maintaining their regular motion trend and "extending forever". According to Fig. 6a,c, the Hurst index shows a convergent trend based on the grouping scheme II. At the same time, the prototype and model follow the same grouping scheme, and the Hurst index variation trend is basically the same, with a value deviation of less than 10%.

### Similar conversion analysis

**Figure 7:**
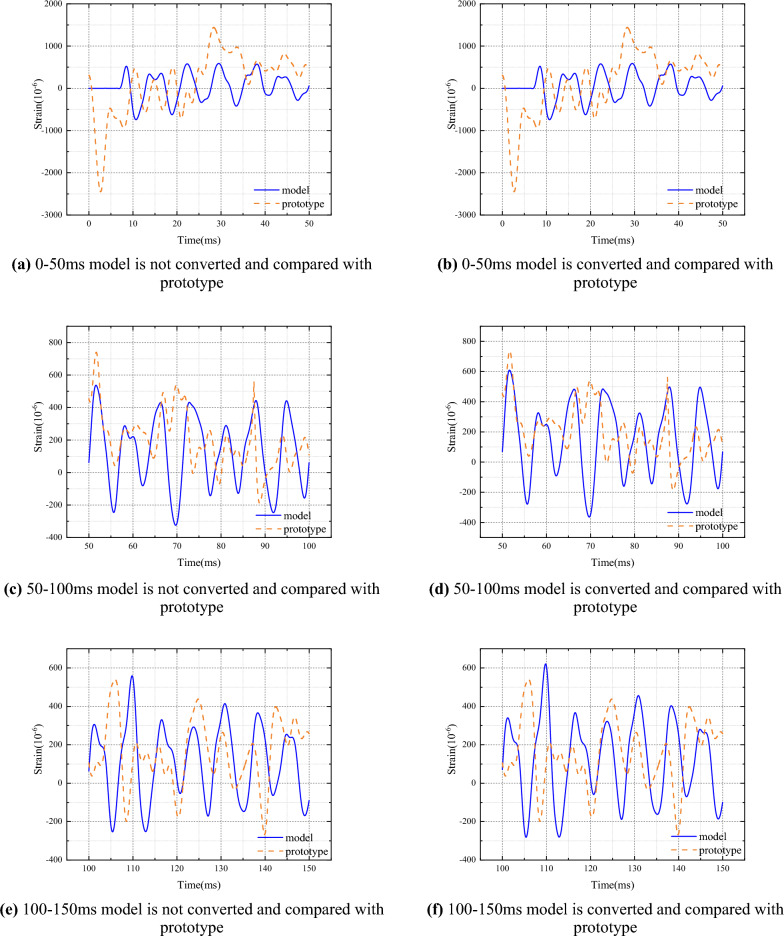

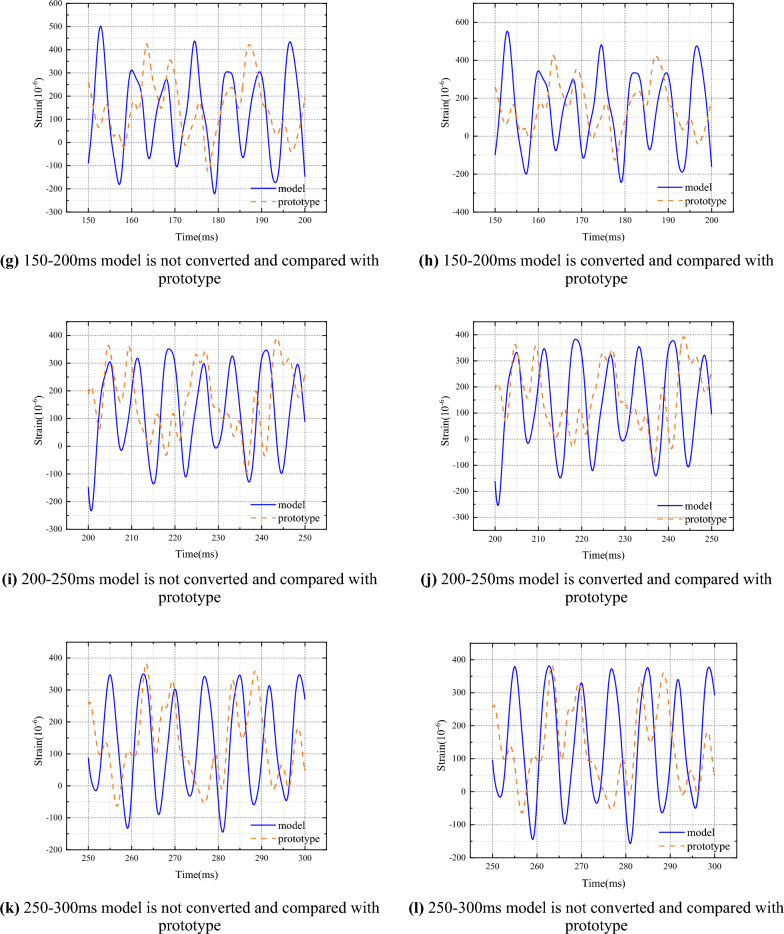
The measured and transformed results of model strain are compared with the prototype respectively.

Similarly, the original strain data of the model is grouped according to the period, and each group is converted using Eq. (17) (the Hurst index is calculated based on the respective period). In order to accurately compare the results of the prototype and the model, a time correction is performed, where the prototype is taken as 634.79ms and the rest of the time is shifted forward by 634.79ms. For comparison purposes, the results of the prototype and the converted model are divided into sections every 50ms, with the first six sections used for comparison segmentation. The conversion results are shown in Fig. 7, which are compared with the prototype. In order to evaluate the conversion effect, the impact response strain values of the prototype ship stiffened plate structure and the model were calculated, and the prototype strain values were compared with the converted model for multi-parameter comparison, including variance, standard deviation, and maximum value. The error of the converted model stiffened plate structure impact response strain relative to the prototype impact response strain is shown in Table 5. According to the calculation results, the error of the variance of the strain value changes from 0.53558 to 0.44191, the error of the standard deviation changes from 0.31851 to 0.25294, and the error of the amplitude changes from 0.59169 to 0.5373 (Table 5).

**Table 5:** Comparison of model strain conversion values with prototype strain measurements for each parameter.

Parameters	Explosion distance	Explosive charges quantity
Variance	0.53558	0.44191
Standard deviation	0.31851	0.25294
Amplitude	0.59169	0.5373

### Verification of simulation results

**Figure 8:**
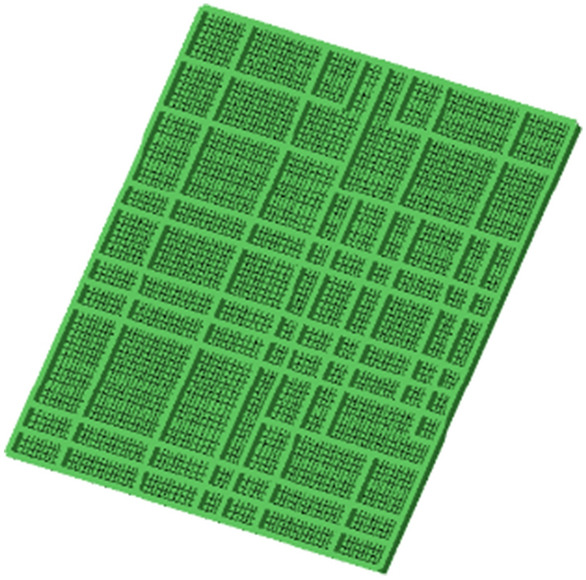
Hull frame model.

Now, take a part of the stiffened plate from a ship and conduct a simulation test on it. The model of the ship's stiffened plate is shown in Fig. 8, and the hull plate frame scaling simulation test conditions is shown in Table 6. The size of the ship's stiffened plate is 18m*17.4m*0.02m (corresponding to the size of a cabin on a real ship), and it is reinforced with T-shaped bars along the horizontal and vertical directions of the ship's stiffened plate^26–28^.

**Table 6:** Hull plate frame scaling simulation test conditions.

Modelling	Length /m	Plate thickness /m	Reinforcement bar size	Equivalent /kg	Margin of error /m	Computation time /s
Prototype	18*17.4	0.02	$\frac{0.012 \times 0.05}{{0.016 \times 0.25}}$	500	30	1
$\lambda =$ 1/2	9*8.7	0.01	$\frac{0.06 \times 0.025}{{0.08 \times 0.125}}$	62.5	15	0.5
$\lambda =$ 1/3	6*5.8	0.0067	$\frac{0.04 \times 0.017}{{0.0053 \times 0.083}}$	18.5	10	0.33
$\lambda =$ 1/4	4.5*4.35	0.005	$\frac{0.03 \times 0.0125}{{0.04 \times 0.0625}}$	7.8125	7.5	0.25
$\lambda =$ 1/5	3.6*3.48	0.004	$\frac{0.0024 \times 0.01}{{0.0032 \times 0.05}}$	4	6	0.2

**Figure 9:**
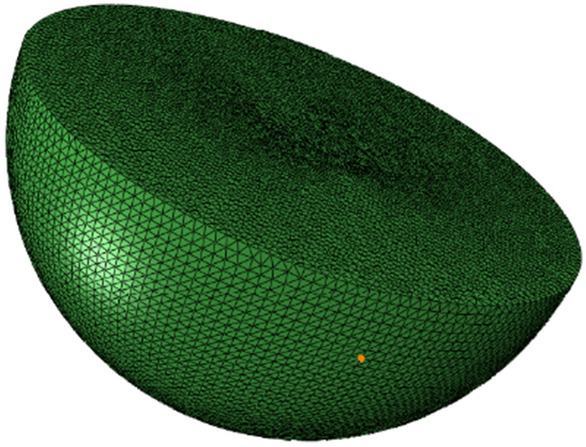
Water area model.

The simulation experiment was conducted based on ABAQUS finite element simulation software. The ship stiffened plate model was divided into 9264 nodes and 9640 elements. The grid division is shown in Fig. 8. In order to ensure that the calculation results are not affected by the number of grids, the number of grids is kept unchanged at each scale; Similarly, set the water area (shock load transfer medium), the total number of nodes: 97,794, the total number of grids: 552,113, the water area is a hemisphere with a diameter of 90m, and set the original explosion shock wave movement direction as the positive direction of the z axis of the corresponding position with the water area center of the upper surface of the z axis of the coordinate system as the origin. The water structure is shown in Fig. 9.

The material properties of the board and frame structure and waters are set as shown in Table 7.

**Table 7:** Material properties.

Character radical	Parametric	Numerical value
Water area	Flexural modulus (N/m^2^)	2.14 × 10^9^
	Densities (kg/m^3^)	1025
Plate frame structure (steel)	Young modulus (Pa)	2.1 × 10^11^
	Poisson ratio	0.3
	Density (kg/m^3^)	7850

**Figure 10:**
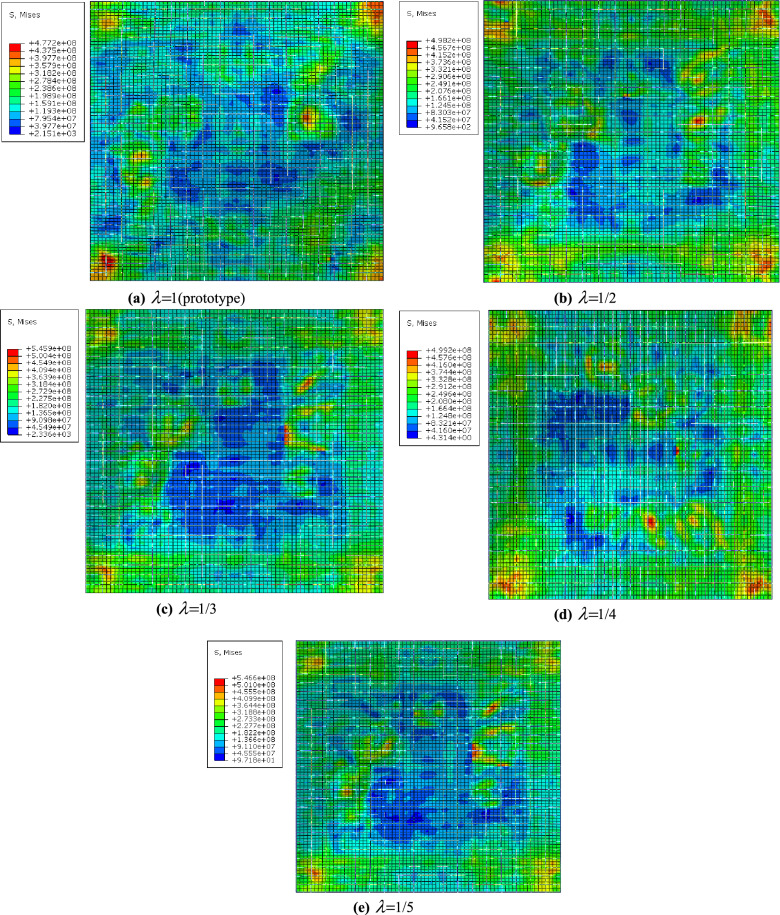
The simulation result of the last calculation time.

The calculation time of the prototype is set to 1 s, and the sampling frequency is 100 kHz, which means that there are a total of 1,000,000 data points in the response process curve. In order to ensure the consistency of the number of sampled data points and eliminate the impact of the difference in the number of sampled data points on the Hurst exponent, under the condition of a scaling ratio of 1/2, the sampling frequency is set to 200 kHz, which will also have 1,000,000 data points. For the remaining scaling conditions, the sampling frequency is also adjusted similarly, so that ultimately 1,000,000 data points are included in the calculation time period.^29^ The shock wave loading pulse width of underwater explosions has a strong nonlinearity^30^, so the Hurst indices of displacement, velocity, and acceleration during the shock wave pulse width time are calculated separately. The prototypes and shock wave pulse widths for each scaling ratio are shown in Table 8 (extracted from the origin in the z direction). At the same time, the calculation results of the Hurst index are shown in Table 8. The simulation result of the last calculation time is shown in Fig. 10.

**Table 8:** Statistics of Hurst index for similar models at different scales.

Reduced scale	Shock wave pulse widt ($\mu s$)	Displacement		Speed		Accelerations	
		Hurst exponent number	Error with prototype	Hurst exponent value	Error from prototype	Hurst exponent value	Error from prototype
Prototype	24	0.698786	–	0.711439	–	0.626169	–
$\lambda = 1/2$	12	0.689262	-1.36%	0.74665	4.95%	0.615858	-1.65%
$\lambda = 1/3$	8	0.692606	-0.88%	0.70909	-0.33%	0.635037	1.42%
$\lambda = 1/4$	6	0.68715	-1.67%	0.707745	-0.52%	0.65439	4.51%
$\lambda = 1/5$	4.8	0.696839	-0.28%	0.714675	0.45%	0.648963	3.64%

To ensure that the model corresponds to the prototype in time and eliminate the influence of small mean square response values, the last data point of the mean square response of the model and prototype is taken to calculate the error between the two. Table 9 presents the mean square response results of prototype and model displacement. The stress simulation results at the corresponding time of the last data point are shown in Fig. 10.

**Table 9:** Prototype and model displacement mean square response results statistics.

Scale ratio	Prototype (mm)	Model raw value	$\frac{1}{{\lambda }^{H}}$	Model conversion value	Error %
$\lambda =1/2$	0.005618	0.004540	1.612458	0.005386	-4.32%
$\lambda =1/3$	0.005618	0.002596	2.155164	0.005207	-7.90%
$\lambda =1/4$	0.005618	0.002112	2.59242	0.006175	9.02%
$\lambda =1/5$	0.005618	0.001817	3.069514	0.006191	9.26%

## Summary

In order to more accurately analyze the similarity law of nonlinear impact response between the model and the prototype ship's stiffened plate structure under explosion impact, improve conversion accuracy, and provide guidance for offshore operations and ship design. This article innovatively uses the Hirst index to describe the similarity law of the nonlinear impact response between the model and the prototype ship's stiffened plate structure under explosive impact, and derives it based on the similarity transformation principle. The specific conclusion is as follows:

1. Design and manufacture ship prototypes and model ship stiffened plate structures, with dimensions that fully conform to a geometric similarity relationship of 1.5:1. Estimate the natural frequencies of the prototype and model using empirical formula method as 55Hz and 80Hz, respectively. The natural frequency of the prototype is about 50Hz, and the natural frequency of the model is about 71Hz. By using the Fourier transform method, the natural frequencies of the prototype and model are 60Hz and 90Hz, respectively. Finally, the sampling periods for the prototype and model are 0.01666s and 0.01111s, respectively.
2. Based on the classical similarity law, the similarity transformation relationship between range R and mean square deviation S is derived, and the Hurst index of the model and prototype are equal. According to the grouping method, the strain data of the prototype and model are grouped according to the sampling period, and the relationship between the Hurst index and time is obtained. The prototype and model follow the same grouping scheme, and the change trend of the Hurst index is basically the same, with an error of less than 10% between the two.
3. Analyze the response mechanism of ship stiffened plates under impact, and establish a similarity transformation relationship between the model and the prototype. Combining the traditional strain formula, convert the strain results of the model experiment within each natural cycle, and compare the conversion results with the prototype experiment results. According to the calculation results, the error of the average strain value changed from 0.53558 to 0.44191, the error of the standard deviation changed from 0.31851 to 0.25294, and the error of the amplitude changed from 0.59169 to 0.5373.
4. In order to verify the correctness of the conclusions under different working conditions, scaled simulation tests were conducted on the ship's stiffened plate structure. The scaling ratios for structural dimensions are set to 1/2, 1/3, 1/4, and 1/5, respectively. The results show that under different scaling ratios, the Hurst index of displacement, velocity, and acceleration within the pulse width time of the shock wave is about 0.7, and the Hurst index errors of the model and prototype for each parameter response are less than 5%. Based on the mutual conversion relationship between the displacement mean square response results obtained from the prototype and the model, the model results are converted to the prototype and the mean square response value is obtained. The error between the prototype and the model is less than 10%.

In summary, this article proves that the Hurst index of the nonlinear impact response of ship stiffened plate structures is equal, and provides a similarity transformation method that can reduce errors. However, this article only focuses on the nonlinear response of ship reinforced plate structures under underwater explosion conditions. The similarity law of nonlinear response under other working conditions still needs further verification.
